# Dead Teeth in the Jaws

**Published:** 1884-12

**Authors:** 


### Editorial.

### DEAD TEETH IN THE JAWS.

*The Medical Record* has contained a number of articles under the above heading. Samuel Sexton, M. D., of the New York Eye and Ear Infirmary, in an article upon "Pain in the Ears Due to Irritation in the Jaws," relates a number of cases of otalgia and inflammation, complicated with dental caries. That diseases of the ear are frequently induced by dental lesions, no intelligent dentist will for a moment doubt. In commenting upon the cases, Dr. Sexton said that "it was believed that since dentistry had become such a popular business, and dead and diseased teeth had been so carefully retained in the jaws, through their influence, especially among the better-to-do, nervous diseases about the head were becoming alarmingly common. The very custom of wearing false teeth in the mouth attached to vulcanite rubber, celluloid, and other plates, was also an evil of vast proportions. Indeed, he sometimes thought that the evil done through ill-advised dentistry was greater than the possible good arising from the work of the more capable dentists."

There is something of truth in this expression of opinion. The question has often been seriously debated whether the medical profession itself, as a whole, taking all schools and classes into consideration, was not productive of more harm than good, and any thinking medical man must admit that it is really a debatable matter. What then? Should medicine be abolished, and all the competent physicians be forced into other avocations because quacks abound? Shall we burn the building to rid it of its vermin? That dentistry is cursed with so many incompetent men is largely the fault of medicine, which refused the overtures of Harris, and Bond, and their compeers, when they proposed that dental chairs should be established in medical schools, and thus make the treatment of dental diseases a direct specialty of medicine. Now that dentistry has, despite this mistaken policy, "become such a popular business," and now that oral diseases are so generally relegated to dentistry, medical colleges are beginning to see a necessity for the step formerly urged upon them in vain, and chairs of oral surgery and oral diseases are being established in most of the leading medical colleges, not to teach *dentistry*, but to give instruction in a large and important class of maladies, so that medical men may be saved from committing the grave errors that constantly fall under the observation of intelligent dentists. There is no class in the community that more sorely needs a little dental information than does the general practitioner. Properly educated dentists almost daily have their hearts wrenched by the knowledge that physicians, who loudly proclaim their desire to keep up the standard of professional ethics, are in the habit of referring their patients to the veriest dental quacks in the neighborhood, passing by the regular graduates and selecting some advertising charlatan, who is quite incapable of saving either a dead or a living tooth. Medicine sadly needs information as to what is good dentistry, and as to what it is within the province of good dentistry to accomplish. This is amply illustrated in the paragraph quoted above—"dead and *diseased* teeth * * carefully retained in the jaws." Does any intelligent physician believe that good dentistry retains *diseased* teeth? It would seem so by the quotation. Dr. Sexton should understand that it is the dentist's business to restore such to a state of health, or, failing in this, to exercise the surgeon's art, and remove them.

But the editor of *The Medical Record*, taking Dr. Sexton's article as a text, proceeds to preach a sermon that is misleading, because it is founded in a misconception. He says: "It would not be strange if, in the course of events, the day would soon come when * * * all teeth without pulps, and hence in process of more or less rapid decay, as well as those which the deposit of tartar, or other cause, had entirely divested of periosteal nourishment, would be promptly condemned as unfit to remain in the jaws—regarded, in fact, as liable * * * * to endanger the general health, through purulent matter discharged into the mouth from alveolar abscesses, to be continuously swallowed for a long time, or, indeed, in some instances, to be absorbed, and thus produce septicæmic poisoning."

This assumes that teeth without pulps cannot be otherwise than in a state of continuous decay; that pyorrhœa alveolaris is incurable, and that good dentists look with indifference upon alveolar abscesses. Pulpless teeth, if properly managed, become as serviceable and as innocuous as any other, and their treatment, with that of the two latter conditions, forms a large part of the practice of every competent dentist. What would be thought of the surgeon who, in every case of abscess of a limb, counseled amputation without even an attempt at a cure? The editor of *The Medical Record* would, if his advice be followed, reduce dentistry to mere mechanical handicrafture, and the same counsels carried into the domain of medicine would leave every other patient to be provided with a wooden leg or arm, while in ophthalmology there would remain little to do save to insert glass eyes. It should be remembered that a pulpless tooth is not deprived of nourishment, and that a diseased peri-dental membrane is as amenable to judicious treatment as is any other tissue of the body. The surgeon does not despair of the reproduction of periosteum when, during operations, a portion is necessarily removed. Why, then, should it be thought a thing incredible that it may ever be restored when a part of it is lost about a tooth?

But he goes on to say: "There has always been a desire to combine the medical art with the mechanics of dentistry, but the medical training usually given the dental apprentice is entirely too superficial to qualify him to treat diseases, whether arising from the state of the teeth or not; in point of fact, his training does not always prevent harm being done to persons who are willing to have placed in the mouth some one or more of the numerous harmful dental appliances of the present day."

A considerable proportion of the dental profession are regular graduates in medicine. Are they lacking in medical training? If so, whose fault is it? Many more are graduates of reputable dental colleges, the most of which have medical men to fill the medical chairs. Is their medical training insufficient? If so, again whose fault is it? There is a class of dentists who unfortunately came into dental practice when medical schools refused to give any facilities for their special study, and dental schools were not founded, or were in their infancy. They have been hard students all their lives, and have had an experience that, in some cases, is worth all the didactic teaching of the schools. Have they had no medical training? The editorial in question is not only largely founded in a misconception of dental practice, but it seems to us very narrow and intolerant in its scope. The old days of a mechanical apprenticeship in dentistry have long gone by, and the law of the State in which *The Medical Record* is published prescribes what shall be the acquirements of a competent dentist. If medical men ignore, or are ignorant of these requirements, and choose for their patients and themselves the unqualified and incompetent, they have no right to accuse good dentistry of having no higher status than that exemplified by their own chosen practitioners.

It is probable that no properly educated and intelligent medical men ever saw graver mistakes committed in the diagnosis and treatment of oral diseases by dentists than have the latter by physicians. Cases of un-erupted teeth, impacted teeth, and buried roots of teeth, have in many cases been treated by physicians for months, under the mistaken impression that they were tumors of—Heaven only knows what class. Dental irritations of the simplest kind have been diagnosed as neuralgias of deep-seated origin, and operations without number have been undertaken or proposed for their relief. Every dentist can relate scores of such cases within his own practice. What then? Does this prove that medical science in its application to oral diseases is a humbug? By no means; but it does incontestibly demonstrate the necessity for the existence of competent oral specialists—in other words, good dentists.

That there is a class of dentists whose almost sole practice consists in the extraction of teeth for the purpose of supplying their loss with artificial plates, is a matter of regret. But they are simply carrying into practice the implied advice of the editor of *The Medical Record*, in removing "teeth without pulps, teeth with sensitive pulps, as well as those which the deposit of tartar or other causes had divested of periosteal nourishment." The propriety of the coupling of mechanical and operative dentistry together has been a vexed question, even among dentists; but it is one that readily settles itself. There is nothing peculiarly incongruous in their connection, nor does the one necessarily unfit the practitioner for the other. With as much propriety might it be urged that the general practitioner of medicine should undertake no cases of surgery, or ophthalmology, or otology. In the large cities, or in a dense population, where there is enough of practice for every specialist, these matters arrange themselves, and the man in general practice refers his surgical cases to the surgeon. So it is in dentistry. The prominent operative and medical dentists either send their mechanical work outside, or employ a mechanical dentist to do it under their direct supervision. But in small villages, in sparsely settled districts, the physician not only makes his own prescriptions, but he must be his own pharmaceutist. He must not alone attend to calls in general medicine, but he must be an obstetrician, a surgeon, an ophthalmologist, and in some cases a dentist. It is no more a reproach to him that he occasionally pulls a tooth than it is that the regular dentist sometimes fashions an artificial denture. Indeed, no one is quite capable of deciding just how and in what manner a plate shall be inserted save the qualified dentist.

We quite agree with the editor when he says: "It is certainly gratifying to note the establishment of instruction in oral surgery in some of the medical schools, and it is to be hoped that this subject will receive the attention its importance demands." But when he further says—"It must not be supposed, however, that 'dentistry' should be taught, for surely this in its broadest sense by no means constitutes oral surgery;"—we raise our hands in astonishment. If dentistry does not include oral surgery, in the name of all the saints at once, what does it include? What kind of a thing does the editor think a dentist should be? He has already inveighed against the ignorance that improperly treats diseased teeth. Would he divest them of the knowledge that they now have, and make mere mechanics of them? But he denounces the incompetence that inserts artificial teeth without a due knowledge of the medical requirements of the case. How shall this be obtained without some medical study? We must confess that we cannot comprehend what he is really driving at, and must conclude that he has either spoken without due consideration, or in the absence of sufficient dental knowledge to enable him to be a competent and impartial judge. We do not believe that "dentistry" should be taught in medical schools, but we do think that physicians should have enough knowledge of dental and oral diseases to be able to diagnose them, and to refer to those best qualified to treat them such as properly fall within the province of the dentist, and it is just that instruction that we are endeavoring to give in the Medical College with which we have the honor to be connected.

We have deemed it within the sphere of our duties to speak thus plainly upon some of the relations of dentistry to medicine, but it is not here that the first antidote has been applied to the articles, the general tone and spirit of which are much more offensive than the express language. John S. Marshall, M. D., J. Morgan Howe, M. D., C. E. Nelson, M. D., and A. W. Harlan, M. D., have all contributed to *The Medical Record* excellent articles upon the subject.

